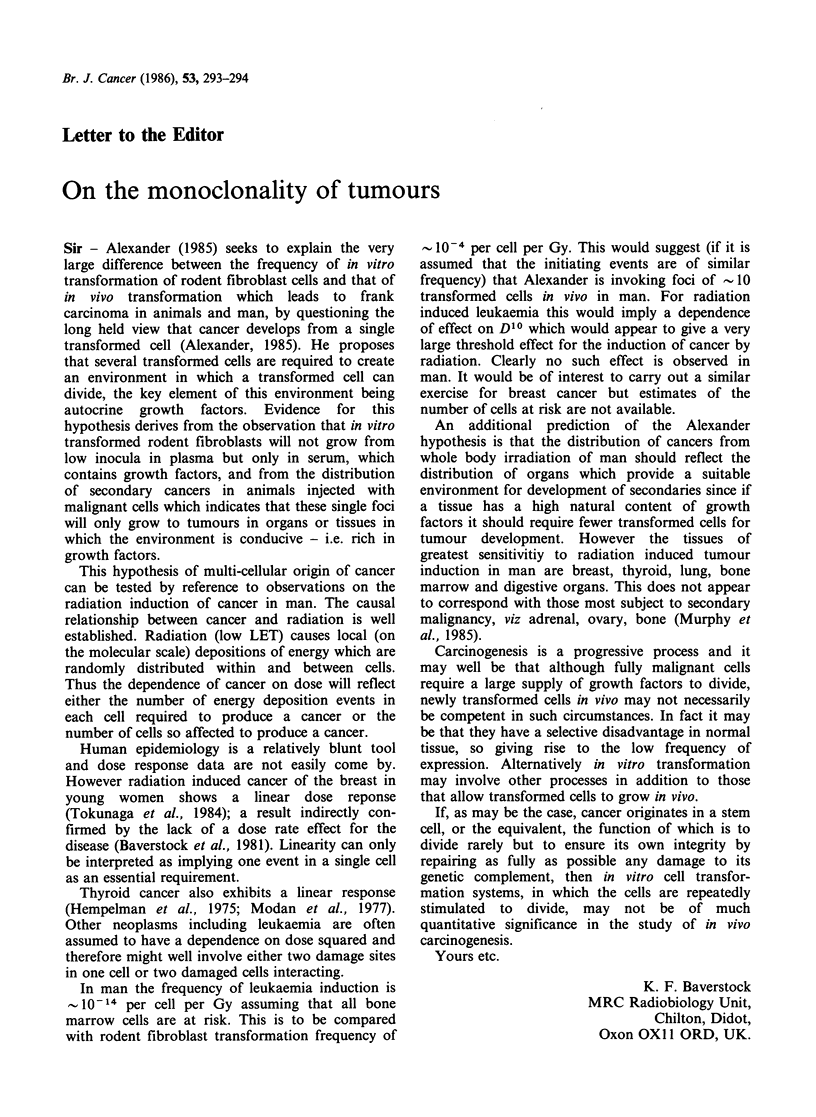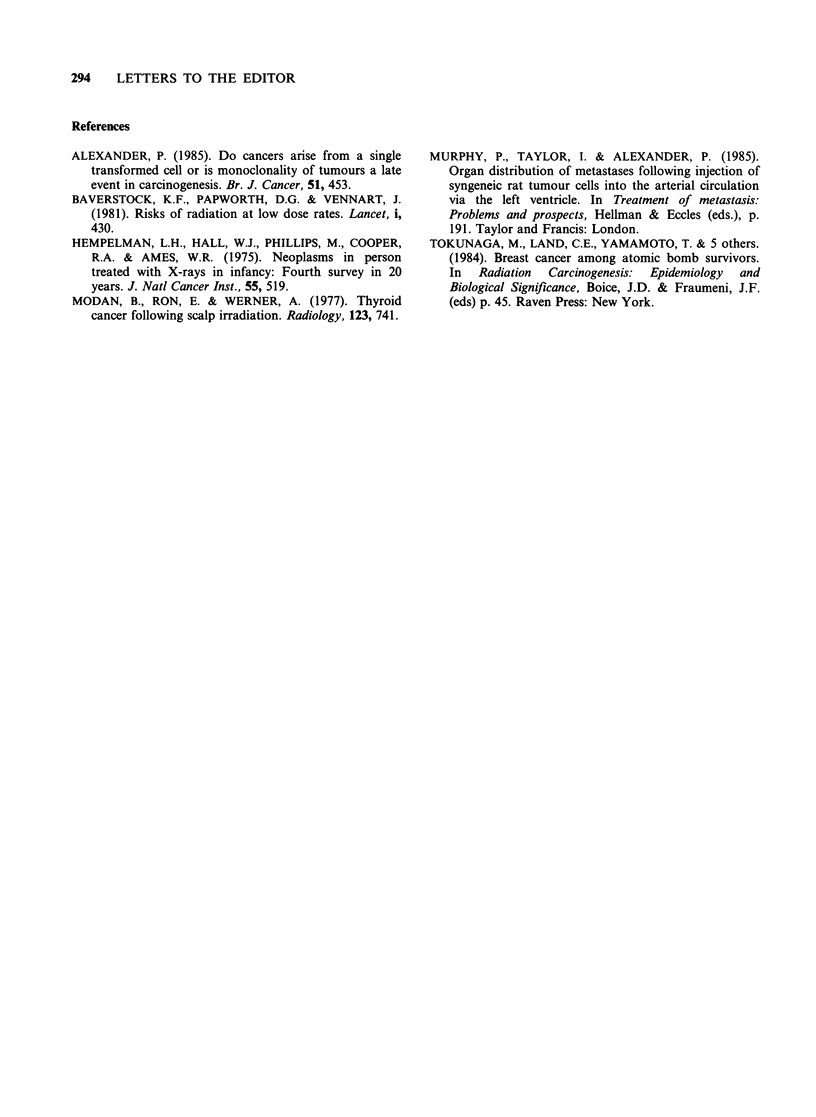# On the monoclonality of tumours.

**DOI:** 10.1038/bjc.1986.49

**Published:** 1986-02

**Authors:** K. F. Baverstock


*Br. J. Cancer* (1986), **53**, 293–294

### Letter to the Editor

### On the monoclonality of tumours

**Sir** – Alexander (1985) seeks to explain the very large difference between the frequency of *in vitro* transformation of rodent fibroblast cells and that of *in vivo* transformation which leads to frank carcinoma in animals and man, by questioning the long held view that cancer develops from a single transformed cell (Alexander, 1985). He proposes that several transformed cells are required to create an environment in which a transformed cell can divide, the key element of this environment being autocrine growth factors. Evidence for this hypothesis derives from the observation that *in vitro* transformed rodent fibroblasts will not grow from low inocula in plasma but only in serum, which contains growth factors, and from the distribution of secondary cancers in animals injected with malignant cells which indicates that these single foci will only grow to tumours in organs or tissues in which the environment is conducive – i.e. rich in growth factors.

This hypothesis of multi-cellular origin of cancer can be tested by reference to observations on the radiation induction of cancer in man. The causal relationship between cancer and radiation is well established. Radiation (low LET) causes local (on the molecular scale) depositions of energy which are randomly distributed within and between cells. Thus the dependence of cancer on dose will reflect either the number of energy deposition events in each cell required to produce a cancer or the number of cells so affected to produce a cancer.

Human epidemiology is a relatively blunt tool and dose response data are not easily come by. However radiation induced cancer of the breast in young women shows a linear dose reponse (Tokunaga *et al.*, 1984); a result indirectly confirmed by the lack of a dose rate effect for the disease (Baverstock *et al.*, 1981). Linearity can only be interpreted as implying one event in a single cell as an essential requirement.

Thyroid cancer also exhibits a linear response (Hempelman *et al.*, 1975; Modan *et al.*, 1977). Other neoplasms including leukaemia are often assumed to have a dependence on dose squared and therefore might well involve either two damage sites in one cell or two damaged cells interacting.

In man the frequency of leukaemia induction is $\sim 10^{-14}$ per cell per Gy assuming that all bone marrow cells are at risk. This is to be compared with rodent fibroblast transformation frequency of $\sim 10^{-4}$ per cell per Gy. This would suggest (if it is assumed that the initiating events are of similar frequency) that Alexander is invoking foci of $\sim 10$ transformed cells *in vivo* in man. For radiation induced leukaemia this would imply a dependence of effect on $D^{10}$ which would appear to give a very large threshold effect for the induction of cancer by radiation. Clearly no such effect is observed in man. It would be of interest to carry out a similar exercise for breast cancer but estimates of the number of cells at risk are not available.

An additional prediction of the Alexander hypothesis is that the distribution of cancers from whole body irradiation of man should reflect the distribution of organs which provide a suitable environment for development of secondaries since if a tissue has a high natural content of growth factors it should require fewer transformed cells for tumour development. However the tissues of greatest sensitivitiy to radiation induced tumour induction in man are breast, thyroid, lung, bone marrow and digestive organs. This does not appear to correspond with those most subject to secondary malignancy, *viz* adrenal, ovary, bone (Murphy *et al.*, 1985).

Carcinogenesis is a progressive process and it may well be that although fully malignant cells require a large supply of growth factors to divide, newly transformed cells *in vivo* may not necessarily be competent in such circumstances. In fact it may be that they have a selective disadvantage in normal tissue, so giving rise to the low frequency of expression. Alternatively *in vitro* transformation may involve other processes in addition to those that allow transformed cells to grow *in vivo*.

If, as may be the case, cancer originates in a stem cell, or the equivalent, the function of which is to divide rarely but to ensure its own integrity by repairing as fully as possible any damage to its genetic complement, then *in vitro* cell transformation systems, in which the cells are repeatedly stimulated to divide, may not be of much quantitative significance in the study of *in vivo* carcinogenesis.

Yours etc.

K. F. Baverstock
MRC Radiobiology Unit,
Chilton, Didot,
Oxon OX11 ORD, UK.

**References**

ALEXANDER, P. (1985). Do cancers arise from a single transformed cell or is monoclonality of tumours a late event in carcinogenesis. *Br. J. Cancer,* **51,** 453.

BAVERSTOCK, K.F., PAPWORTH, D.G. & VENNART, J. (1981). Risks of radiation at low dose rates. *Lancet,* **i,** 430.

HEMPELMAN, L.H., HALL, W.J., PHILLIPS, M., COOPER, R.A. & AMES, W.R. (1975). Neoplasms in person treated with X-rays in infancy: Fourth survey in 20 years. *J. Natl Cancer Inst.,* **55,** 519.

MODAN, B., RON, E. & WERNER, A. (1977). Thyroid cancer following scalp irradiation. *Radiology,* **123,** 741.

MURPHY, P., TAYLOR, I. & ALEXANDER, P. (1985). Organ distribution of metastases following injection of syngeneic rat tumour cells into the arterial circulation via the left ventricle. In *Treatment of metastasis: Problems and prospects,* Hellman & Eccles (eds.), p. 191. Taylor and Francis: London.

TOKUNAGA, M., LAND, C.E., YAMAMOTO, T. & 5 others. (1984). Breast cancer among atomic bomb survivors. In *Radiation Carcinogenesis: Epidemiology and Biological Significance,* Boice, J.D. & Fraumeni, J.F. (eds) p. 45. Raven Press: New York.